# Identification of 24 new microsatellite loci in the sweat bee *Lasioglossum malachurum* (Hymenoptera: Halictidae)

**DOI:** 10.1186/s13104-017-3089-4

**Published:** 2017-12-19

**Authors:** Paul J. Parsons, Christelle Couchoux, Gavin J. Horsburgh, Deborah A. Dawson, Jeremy Field

**Affiliations:** 1 Centre for Ecology and Conservation, University of Exeter, Penryn Campus, Cornwall, TR10 9EZ UK; 2 NERC Biomolecular Analysis Facility, Department of Animal and Plant Sciences, University of Sheffield, Western Bank, Sheffield, S10 2TN UK

**Keywords:** Halictidae, Microsatellite, *Lasioglossum malachurum*, *Lasioglossum calceatum*, Sweat bee

## Abstract

**Objective:**

The objective here is to identify highly polymorphic microsatellite loci for the Palaearctic sweat bee *Lasioglossum malachurum.* Sweat bees (Hymenoptera: Halictidae) are widespread pollinators that exhibit an unusually large range of social behaviours from non-social, where each female nests alone, to eusocial, where a single queen reproduces while the other members of the colony help to rear her offspring. They thus represent excellent models for understanding social evolution.

**Results:**

24 new microsatellite loci were successfully optimized. When amplified across 23–40 unrelated females, the number of alleles per locus ranged from 3 to 17 and the observed heterozygosities 0.45 to 0.95. Only one locus showed evidence of significant deviation from Hardy–Weinberg equilibrium. No evidence of linkage disequilibrium was found. These 24 loci will enable researchers to gain greater understanding of colony relationships within this species, an important model for the study of eusociality. Furthermore, 22 of the same loci were also successfully amplified in *L. calceatum*, suggesting that these loci may be useful for investigating the ecology and evolution of sweat bees in general.

## Introduction

Sweat bees (Hymenoptera: Halictidae) are widespread pollinators which exhibit an unusually large range of social behaviours from non-social, where each female nests alone, to eusocial, where a single queen reproduces while the other members of the colony help to rear her offspring [1]. Sweat bees are also unusual in that social and non-social species are often closely related, with multiple evolutionary transitions having occurred between sociality and non-sociality [2]. Sweat bees thus represent excellent models for understanding social evolution [1, 2]. Here we present a new set of microsatellite loci developed from *Lasioglossum malachurum* (Kirby, 1802), a haplodiploid eusocial species that has been particularly well studied, mainly because it is widely distributed in the Western Palaearctic and because it often occurs in large, dense nesting aggregations that facilitate behavioural research [3–6]. Microsatellite markers are widely used in social evolution research, for example to investigate population structure, estimate genetic relatedness and assign offspring to parents [7–9]. Microsatellite loci have been developed for this species previously [3, 10] but most of them have comparatively low heterozygosities and are difficult to combine into multiplex reactions because of highly specific annealing temperatures and polymerase chain reaction (PCR) mixes. Here, we report 24 new microsatellite markers developed for *L. malachurum*, 14 of which have been efficiently amplified in two multiplex sets. These markers should substantially aid future studies on sweat bee behaviour and ecology.

## Main text

*Lasioglossum malachurum* females were sampled from a field site at Denton in East Sussex, UK in 2015. Genomic DNA was extracted from head, abdomen and/or legs using an ammonium acetate extraction method [11, 12]. DNA concentration was quantified using a Fluostar Optima fluorimeter and its quality assessed using gel electrophoresis. DNA from one foundress (female M4) from Denton was digested using *MboI* and the fragments enriched for dinucleotide and tetranucleotide repeat motifs (following [13]). An Illumina paired end library was then compiled using this repeat-enriched genomic DNA. The NEBNext Ultra library preparation kit (New England Biolabs Inc. Cat. No. E7370S) protocol was followed and DNA sequencing was conducted using a MiSeq Benchtop Sequencer (Illumina). Primer sets were designed from 53 microsatellite sequences using PRIMER3 v0.4.0 [14]. Sequences were confirmed to be unique using BLAST software [15].

Each 2 µl PCR contained approximately 10 ng of air-dried genomic DNA, 0.2 µM of each primer and 1 µl QIAGEN Multiplex PCR mix (QIAGEN Inc. Cat. No. 20614) following [16]. As we required loci that could be reliably multiplexed together for efficient use we designed primers with very similar melting temperatures (± 2 °C) enabling these to be amplified at the same annealing temperature (57 °C). The following PCR profile was used: 95 °C for 15 min, followed by 44 cycles of 94 °C for 30 s, 57 °C for 90 s, 72 °C for 90 s and finally 60 °C for 30 min. PCR amplification was performed using a DNA Engine Tetrad ^®^Thermal Cycler (MJ Research, Bio-Rad, Hemel Hempstead, Herts, UK). PCR products were genotyped on an ABI 3730 48-well capillary DNA Analyser using the LIZ size standard (Applied Biosystems Inc. Cat. No. 4322682). Alleles were scored using GENEMAPPERv3.7 software (Applied Biosystems Inc.). Of the 53 markers, 24 could be scored reliably across the test sample (23–40 females all from the same field site at Denton) (Table 1). The remaining 29 were found to be either monomorphic or unreliable following our PCR methodology (Table 2). It is possible that with more specific optimization, some of these could be used in future studies. We successfully incorporated 14 of the optimized markers into two multiplex panels (using the above PCR reagents and concentrations) with no dropout or artifacts produced (Table 1).

**Table 1:** Characterisation of 24 new *L. malachurum* microsatellites

Locus name	GenBank sequence accession number	Panel and dye	Repeat motif	Primer sequence (5′–3′)	N^a^ tested	N alleles	Expected allele size^b^, size range	HObs	HExp	HWE *p* value	Est. F (null)	L. cal success^c^
Lma02	MG273262	1	(TC)_13_	F: CCGAGTTCATCAACATCCTC	23	10	150	0.87	0.83	0.712	− 0.037	P
		NED		R: TTGATTATCAGCGAGATGAGC			139–185					
Lma03	MG273263	1	(AG)_14_	F: AAAGCGTTGCGAGACACC	38	7	154	0.816	0.745	0.103	− 0.063	P
		PET		R: AGCATAATGGAAACCCAACG			137–167					
Lma04	MG273264		(TG)_12_	F: CGTTACCGCGTTGGTTTC	37	6	169	0.649	0.727	0.162	0.034	M
				R: GTCTTGTCTAACCGCAACAGC			165–177					
Lma12	MG273265	2	(CT)_12_	F: CCAACCGAACACCAACTTTC	39	10	150	0.667	0.701	0.413	0.017	P
		PET		R: CTCCCGGGTTGTCATGTAAG			131–181					
Lma14	MG273266	1	(AG)_14_	F: CAACGCGTGACAGGTGATAC	40	14	170	0.825	0.896	0.186	0.035	P
		6-FAM		R: CGGCTACGTTCCACTATGAAG			162–192					
Lma20	MG273267		(AG)_19_	F: AGCGCTCGATGACTGTCG	39	17	210	0.872	0.889	0.087	0.007	F
				R: TTGCGCAAGCCGTTCTAC			196–262					
Lma21	MG273268	2	(GA)_16_	F: CGGTAAACTTGCTTCGACCTG	38	11	137	0.868	0.85	0.053	− 0.026	F
		NED		R: CCGATTCCTTCACAGACACG			135–156					
Lma23	MG273269		(GA)_13_	F: GATAATCAATGGTAATCGGTTGG	40	11	167	0.85	0.838	0.179	− 0.017	M
				R: TTAACATCGTTCGCTTCTCG			154–218					
Lma24	MG273270	2	(GA)_13_ CA (GA)_6_	F: TCCTCGGACAAGGAGATACG	40	13	172	0.925	0.891	0.723	− 0.026	P
		6-FAM		R: TTCGGGTACCGTTCAGTCTC			141–181					
Lma27	MG273271		(GA)_13_	F: GCTGGCAGCTCTGGAGAAG	38	9	189	0.737	0.804	0.071	0.032	P
				R: TGACGGCCATTTAGTTCGTC			177–199					
Lma29	MG273272	1	(CT)_4_ TT (CT)_9_	F: CTCGTCCCTCGTGTGACTC	38	12	204	0.868	0.883	0.725	0.003	P
		PET		R: GTATCGTGCGTGCGTGTC			201–231					
Lma30	MG273273		(GACGA)_6_	F: TCCGTCTCTGGTCGATACTG	38	3	237	0.447	0.407	0.854	− 0.075	P
				R: ACAGCAGCATCTGAACTTGC			225–235					
Lma31	MG273274		(TCTT)_10_	F: CGCACTCCGCTTTTCCTC	40	6	146	0.55	0.664	0.049	0.084	P
				R: CGTCACCAGGAGAGCAAGG			142–164					
Lma34	MG273275		(CT)_12_	F: TCTGAACAGTACGGAACAATGC	40	6	176	0.675	0.684	0.718	− 0.009	P
				R: ACCGACACGGGAGAGAGAG			165–179					
Lma36	MG273285	1	(CT)_16_	F: GGCCCTTCGACTTTGTTG	38	8	188	0.737	0.785	0.298	0.027	P
		VIC		R: GAATCTCTGGGTGCTCTAACG			185–199					
Lma39	MG273276	2	(CTAT)_8_	F: CGAGCCTATGCAGAGAACAG	38	7	205	0.789	0.75	0.68	− 0.034	P
		PET		R: TGGATGGCTGCTGAGTAAAC			205–237					
Lma40	MG273277	2	(GA)_12_	F: CGTTCGTTCGTTCGTTACTG	38	14	150	0.947	0.906	0.74	− 0.029	P
		VIC		R: CAGAGTGCGTCGCTTGTTAG			155–189					
Lma42	MG273278		(AG)_13_	F: ACCATCGCCCTTCCACTAC	40	5	167	0.75	0.733	0.623	− 0.016	P
				R: CCGAAACTATTCGCCCATC			161–169					
Lma48	MG273279	2	(TC)_14_	F: GTTGGATGCATCTGGAGGAC	38	6	206	0.763	0.722	0.14	− 0.043	M
		NED		R: TGCGGTGGTTATTGATTTCC			193–209					
Lma49	MG273280		(GAAA)_10_	F: GAGAGGGTGGTTGCACTACG	38	4	209	0.684	0.62	0.786	− 0.055	M
				R: CTCGTGGAATCGAACTCACC			189–209					
Lma50	MG273281		(CT)_3_ CG (CT)_12_	F: CGTTTAACCGGCTCGCTAC	38	8	181	0.684	0.763	0.726	0.051	P
				R: CCGCGAATAAGTGGAGTGTC			163–209					
Lma51	MG273282	1	(CT)_11_	F: GAGAAATTGCCAGCAAACATC	40	4	243	0.475	0.545	0.259	0.066	P
		6-FAM		R: AGTTTCGTGGAAGGGAACG			237–243					
Lma52	MG273283	1	(TG)_11_	F: CGGCAACTGCTTGCATAAC	40	5	156	0.8	0.732	0.575	− 0.056	M
		VIC		R: CCCGTAGCACTCGCATACTC			151–159					
Lma53	MG273284	1	(AC)_12_	F: ACGCGGGATTACTTTCAATC	40	9	228	0.675	0.759	0.053	0.057	P
		NED		R: CCAATTATCGGGTGAAGGAG			217–241					

^a^N: number of diploid, unrelated *L. malachurum* females genotyped (all from the same population at Denton)

^b^Based on the sequenced individual (sample M4); Hobs and HExp: observed and expected heterozygosities; HWE: *p* value when testing for deviation from Hardy–Weinberg equilibrium; F(Null): Estimated frequency of null alleles

^c^Amplification success across 14 *L. calceatum* individuals: *F* failed to amplify, *M* monomorphic, *p* polymorphic

**Table 2:** Identification of a further 29 markers that were rejected and not considered for multiplex panels

Locus name	GenBank sequence accession number	Repeat motif	Primer sequence	Expected allele size^†^	Reason for dropping (tested in 23–24 individuals)
Lma01	MG273287	(TGAC)_7_	F: AACGCCTCGGTGAACCTG	108	Monomorphic
			R: TCGAGTTCTCCCTCCTCGTATC		
Lma05	MG273288	(TTTC)_7_	F: ATGCGTCTAAATCGTTCCTG	178	Monomorphic
			R: AACAAAGAATGAACGAACGTG		
Lma06	MG273289	(AG)_11_	F: CGGGAACGACGGAGAGAG	184	False peaks
			R: ACGGGTCTGTTCACCCTTTG		
Lma07	MG273286	(GAAA)_5_	F: GTCATGGAGAGGGTGGTTG	189	No product
			R: CAATCTCAACCGTGTTCGTC		
Lma08	MG273290	(TTCT)_7_	F: CTATCCGAGGCCTGTACACTG	192	No product
			R: ATCTGAAATCGTGGCTGGTC		
Lma09	MG273291	(AGAA)_5_	F: ACGGGACTGAAAGGGACAC	201	Monomorphic
			R: TACTTCGCGTGCCTGTCTC		
Lma10	MG273292	(AAAG)_7_	F: GAGACAACGAGGGAGAAAGC	206	Stutter
			R: AACCTCAACCGTGTTCGTC		
Lma11	MG273293	(GA)_7_	F: CTTGTACCACGCGTACACACC	111	False peaks
			R: GCCCTGCGTCTTCTCCTC		
Lma13	MG273294	(CT)_18_	F: GCTCATCGAGGACGAGGTG	154	False peaks
			R: GCGGTTGGCTGTCATAAGTG		
Lma15	MG273295	(TGCT)_5_	F: GGACAGTCCGACGAAGGAG	179	No product
			R: GCTTCATCCCTTTACTCCATAGC		
Lma16	MG273296	(TC)_20_	F: ACATTGTTCACCGGACAAATC	187	Monomorphic
			R: CGTCGAGGATAAGGTTACGG		
Lma17	MG273297	(TC)_11_	F: GTCAACGGTAATCCGAGGTG	189	False peaks/Stutter
			R: TGATACACCGGGAACCATTC		
Lma18	MG273298	(AG)_16_	F: GGGATACTAGACAGCCGGAATATAG	193	False peaks
			R: GAATGAACCACGCCGAAG		
Lma19	MG273299	(TC)_20_	F: TGTAAACGGCCGAAGTGTC	203	False peaks/Stutter
			R: ACAATGTGTGTTCCGGTCAG		
Lma22	MG273300	(TCTT)_5_	F: GCCGGACCAGATTAAATGC	151	No product
			R: AAAGACGAGGCTCAAAGAAGC		
Lma25	MG273301	(CTAT)_6_	F: CGAAATACCGTTAACCAACATC	180	Monomorphic
			R: TAAAGTGGCGAGTGATGGAC		
Lma26	MG273302	(AG)_16_	F: CTTCGATTCCTCGGGTCAC	188	No product
			R: TTCCGGCACGTTTATGTAGC		
Lma28	MG273303	(CT)_17_	F: ATTCGCGACAATGAACGAG	193	False peaks
			R: CAAACGCGAGTCAATAAATCC		
Lma32	MG273304	(TC)_16_	F: CGACGTACCTCTGCTTCCTC	152	Stutter
			R: AGGTCACTTAAATGGTGGTTGG		
Lma33	MG273305	(GA)_19_	F: CTCTTCTCGATTCCGTCTGG	167	False peaks
			R: TTTCGGCTCTTTGCTCTCTC		
Lma35	MG273306	(GAGT)_5_	F: CCTTCGAGAGGTCAGAGCTAAAG	181	No product
			R: CACGTGGCACCACAAATTC		
Lma37	MG273307	(TTCT)_5_	F: GTGGCCTATGCTCCTCTCC	190	Monomorphic
			R: ATCTGAAATCGTGGCTGGTC		
Lma38	MG273308	(GACA)_9_	F: AGAGACAAAGGCGGAGACAG	197	False peaks/Stutter
			R: TATCTGCGAGACCGACGA		
Lma41	MG273309	(TC)_20_	F: AATGATTGTGAACAGTTTGGTATG	152	Stutter
			R: CGAGACTGCAAGAAGTTTCAC		
Lma43	MG273310	(AG)_17_	F: TTCAGCCGAGGGTAGCAC	178	False peaks
			R: CGTACCATCATCTCGTGTCG		
Lma44	MG273311	(AG)_15_	F: ATGAGACTGGCACGACTGTG	182	False peaks
			R: ATGCGTCGCTCCCTTAATC		
Lma45	MG273312	(CT)_15_	F: TTTCGCATCCATCTTCCTTC	189	False peaks/Stutter
			R: CGCGAATTTCGGTATCTTTC		
Lma46	MG273313	(TCCT)_5_	F: TCCCTTTACCTTCCTTTCTCG	190	Monomorphic
			R: TGCAACATTTGTACCGAACAG		
Lma47	MG273314	(CTTT)_5_	F: CTATCCGAGGCCTGTACACTG	197	Stutter
			R: GGGTAAGCAAGCATCGTTTC		

^†^ Based on the sequenced individual (sample M4)

The numbers of alleles and heterozygosities were calculated for each of the 24 loci using CERVUS v3.0.6 and with the sample sizes shown in Table 1 [17]. Tests for deviation from Hardy–Weinberg equilibrium (HWE) and linkage disequilibrium (LD) were conducted using GENEPOP web version 4.2 [18]. To correct p-values in multiple tests, the Q Value was applied to LD p-values. The q value is a measure of the significance in terms of false discovery rate, rather than conventional Bonferroni correction which attempts to measure significance in terms of false positives only [19]. Observed levels of heterozygosity ranged from 0.45 to 0.95 with 3–17 alleles per locus (Table 1). Only *Lma31* deviated from HWE (*p* = 0.049). No groups of loci displayed LD, providing no evidence of physical linkage based on the individuals genotyped.

These loci are likely to be useful for investigating the ecology and behaviour of *L. malachurum* and also potentially that of other sweat bees. Indeed, we have successfully amplified 22 of the 24 loci in *L. calceatum* (Scopoli) individuals sampled in the UK; only *Lma20* and *Lma21* failed to amplify and 17 of the 22 loci that did amplify were polymorphic (Table 1; Davison & Field, in prep.).

## Limitations

Due to the relatively short read length of the MiSeq Benchtop Sequencing system we were unable to design primer sets to amplify greater than 300 bases. This may however be somewhat fortuitous; the incorporation of larger markers into multiplex panels often proves problematic, since they are generally harder to amplify than markers with smaller products and are more susceptible to dropout [20].

## Abbreviations

HWE: Hardy–Weinberg equilibrium
LD: linkage disequilibrium
PCR: polymerase chain reaction
